# Chlorogenic Acid Enhances Doxorubicin-Mediated Cytotoxic Effect in Osteosarcoma Cells

**DOI:** 10.3390/ijms22168586

**Published:** 2021-08-10

**Authors:** Alessia Salzillo, Angela Ragone, Annamaria Spina, Silvio Naviglio, Luigi Sapio

**Affiliations:** Department of Precision Medicine, University of Campania Luigi Vanvitelli, Via L. De Crecchio 7, 80138 Naples, Italy; alessia.salzillo@unicampania.it (A.S.); angela.ragone@unicampania.it (A.R.); annamaria.spina@unicampania.it (A.S.); luigi.sapio@unicampania.it (L.S.)

**Keywords:** chlorogenic acid, doxorubicin, osteosarcoma, apoptosis, p44/42 MAPK

## Abstract

Despite the recurring outbreak of resistance mechanisms and adverse reactions, doxorubicin (Doxo) still remains the standard-of-care for several cancers, including osteosarcoma (OS). As an appealing source of phytochemical compounds, naturally occurring molecules have extensively been reported to overcome Doxo limitations in preclinical models. Unlike other dietary polyphenols, only few studies recognize chlorogenic acid (CGA) as a potential partner in combination therapy, while, conversely, its anticancer evidence is steadily growing, ultimately in OS. On this basis, herein we examine the cooperating effects between CGA and Doxo in U2OS and MG-63 human OS cells. With respect to Doxo alone, the concomitant administration of CGA further decreased cell viability and growth, promoting cell death potentially via apoptosis induction. Furthermore, a longer-lasting reduction in clonogenic potential deeply supported the CGA ability to improve Doxo efficacy in those cells. Remarkably, CGA treatment ameliorated Doxo-induced cytotoxicity in H9c2 rat cardiomyocyte cells instead. Although inactivation of p44/42 MAPK was detected in response to CGA plus Doxo, PD98059-mediated p44/42 MAPK impairment enhanced the combination outcome in OS cells. These findings firstly propose CGA as a promising chemosensitizer and cardioprotective agent in OS therapy, suggesting the p44/42 MAPK pathway as relevantly involved in CGA-mediated Doxo susceptibility.

## 1. Introduction

While it is undeniable that more innovative and accurate pharmacological approaches are constantly tested in preclinical cancer models, even exhibiting promising results, chemotherapy still represents the only partially effective treatment for certain tumor types, as well as tumor stages, in clinical practice [1].

Even though this class of medications displays a high response rate in first-line regime, prolonged administration is often associated with resistance and side-effect occurrence [2,3]. Such outcomes are especially prominent when anthracyclines are chronically employed in managing high-grade and metastatic malignancies [4].

As the most extensively used anthracyclines, doxorubicin (Doxo) is currently approved to treat several neoplastic disorders including breast and gynecologic tumors, lymphoma, and lung cancer [5]. Moreover, Doxo-based chemotherapy also constitutes the standard care in osteosarcoma (OS), the most frequent primary bone-related tumor [6]. Disrupting both DNA synthesis and replication, Doxo induces cancer cell death in multiple ways, which mainly affect topoisomerase II poisoning and DNA base pair intercalation [7].

Just as with other cancers, even in OS, the successful rate in first-line is usually followed by a dramatic decline in responsiveness and collateral effects, which impede a further dose intensification [8]. Cardiovascular toxicity represents instead one of the major and potentially lethal Doxo-induced side effects in both cancer patients and survivors, suggesting the existence of such a memory mechanism in cardiac cells [9]. As an oxidant agent, Doxo promotes free radical production in myocardiocytes, which in turn disrupts contractile muscle integrity and sinus rhythm [10].

Although numerous attempts have been made in order to identify novel and more effective approaches, OS prognosis still remains very poor with a 5-year survival ranging from 20% to 70% [11].

In the last decades, combination therapy has received great attention as potential successful strategy to overcome resistance and mitigate undesirable toxicity [12]. Complying these requirements, natural molecules have been selected as suitable partners in chemotherapy, thus becoming an attractive source of chemical compounds [13,14].

Whilst from a chemical point of view chlorogenic acids (CGAs) originally described a heterogeneous group of ester compounds that result from the binding between trans-cinnamic acid and quinic acid, this term currently identifies a unique isoform of caffeoylquinic acid, namely 3-caffeoyl quinic acid (3-CQA) [15].

Due to its natural abundance in different plant-related foods, such as coffee beans, eggplant, artichoke, sunflower seed kernels, and fruit, 3-CQA, or 5-CQA according to the recent IUPAC guidelines, has extensively been investigated in living systems [16].

Despite the existence of controversial evidence in safety perspectives, which include absorption, bioavailability, and excretion, multiple pharmacologic properties and biological effects have been ascribed to CGA [17]. Additionally, an increasing number of compelling studies draw the attention to the CGA ability to mitigate various harmful conditions such as metabolic syndrome, hepatic steatosis, gastrointestinal dysfunctions, inflammatory stress, and cardiovascular diseases [15,16].

Similarly, CGA pharmacological administration, as well as food supplementation, has been reported to reduce tumorigenicity in a large number of cell-based and animal cancer studies [18]. Besides affecting cancer cell growth and death, CGA has also been proposed as chemopreventive agent thanks to its ability to delay or counteract the occurrence of chemical carcinogenesis [19]. More recently, CGA-mediated antitumor properties have been described in renal cell carcinoma, glioblastoma, and non-small cell lung cancer [20,21,22]. In this connection, we lastly examined the CGA consequences in three different human OS cell lines, demonstrating that, albeit to a different extent, CGA inhibits growth and promotes cell death via apoptosis induction [23]. Moreover, CGA prevented Tyr705 Signal Transducer and Activator of Transcription 3 (STAT3) phosphorylation and triggered a remarkable Extracellular-signal-Regulated Kinase1/2 (ERK1/2) activation [23,24].

Strictly, despite an ever-growing number of findings support the anticancer role of CGA in preclinical models, very limited studies propose this compound as a chemotherapy partner and, among them, just two investigate the CGA involvement in Doxo susceptibility, recognizing conflicting results and leaving this choice inconclusive [25,26,27,28].

Taking into account the mentioned state-of-the-art, this study has been conceived with the purpose of assessing potential cooperating effects in pharmacodynamics between CGA and Doxo in OS models. Specifically, using multiple methodological approaches, aimed at defining the impact on cell growth and death, we compare combination effectiveness with individual treatments. Finally, characterization of the biological effects, as well as the molecular mechanisms, involved in CGA-mediated Doxo sensitization are also addressed.

## 2. Results

### 2.1. CGA and Doxo Affect Cell Viability in OS Cells Differently

Before addressing the potential impact of combination treatment CGA plus Doxo in OS models, we preliminary tested the outcomes of single drug administration on those cells. Specifically, using U2OS and MG-63 cells as widely accepted representative human OS models, we separately assessed the metabolic activity in reaction to CGA and Doxo as an indirect measure of cell viability [29].

**Figure 1:**
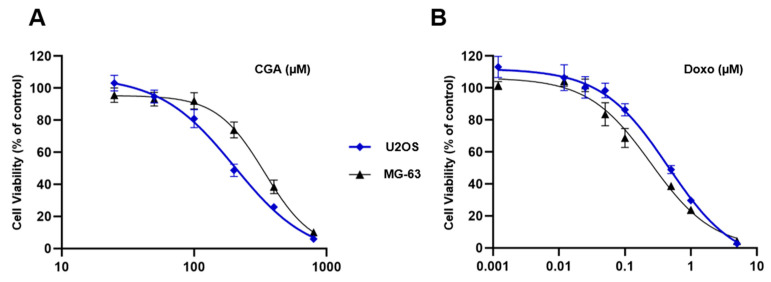
Evaluation of CGA and Doxo-mediated effects in OS cells. U2OS and MG-63 were treated with increasing concentrations of CGA (from 25 to 800 μM) (**A**) and Doxo (from 0.001 to 5 μM) (**B**) for 72 h. Cell viability was estimated by MTT and shown in figure as average rate ± standard deviation (SD) relative to the control of three independent experiments.

In accordance with our previous findings [23], Figure 1A shows a different responsiveness to CGA between U2OS and MG-63, the latter recognized as the least sensitive model. These discrepancies became quite obvious especially when OS cells were exposed to 200 and 400 µM CGA for 72 h. Indeed, whilst at these doses the inhibition index was 52% and 74% in U2OS, respectively, in MG-63, the losses were partially restrained, achieving values of 27% and 62%, correspondingly.

A different matter concerns the Doxo ability to reduce cell viability between U2OS and MG-63. Although no dissimilarities were observed in dose–effect curves shape, tiny differences in mitochondrial susceptibility were detected (Figure 1B).

Overall, these data suggest an effective yet different ability to respond to CGA and Doxo between the investigated OS models.

### 2.2. Combination CGA plus Doxo Greatly Impairs Cell Viability and Growth in OS Cells

Leveraging the above results, we selected three effective concentrations of both CGA (100, 200, and 400 μM) and Doxo (0.05, 0.1, and 0.2 μM), and tested their impact on cell viability with the purpose of assessing potential cooperating effects between these two compounds. In this respect, U2OS and MG-63 were exposed for 48 h to single and combinatory treatments, mixing CGA and Doxo with a constant dilution ratio.

**Figure 2:**
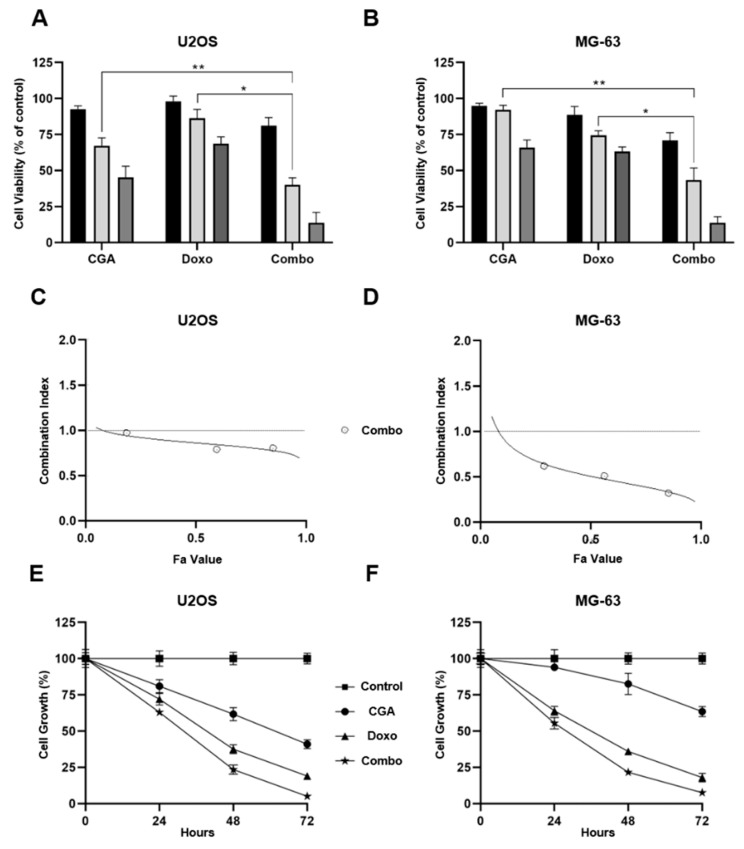
Assessment of CGA, Doxo, and CGA plus Doxo-mediated effects on cell viability and proliferation in OS cells. 100 μM (black bar), 200 μM (light-gray), and 400 μM (gray) of CGA and 0.05 μM (black bar), 0.1 μM (light-gray), and 0.2 μM (gray) of Doxo were administrated individually and together in U2OS (**A**) and MG-63 (**B**) for 48 h. Subsequently, cell viability was estimated by MTT assays and shown as mean ± SD in percentage of control. U2OS (**C**) and MG-63 (**D**) Fa-CI plots were determined employing CompuSyn software analysis. Representative reports are illustrated in figure. Growth curves at 24, 48, and 72 h were recorded in response to untreated (control), 200 μM CGA, 0.1 μM Doxo, and CGA plus Doxo (combo) in both U2OS (**E**) and MG-63 (**F**). Percentage growth values of three distinct biological replicates, expressed as average ± SD, are displayed. * *p* < 0.05, ** *p* < 0.01 by an unpaired two-tailed *t*-test.

Figure 2A displays a clear dose–response relationship between the CGA concentrations and cell viability inhibition in U2OS, as well as in response to Doxo. However, even more interestingly, co-administration of CGA plus Doxo further decreases cellular metabolic activity compared to single treatments, suggesting an additional reduction in viability. Although already obvious at even lower doses, 200 μM CGA plus 0.1 μM Doxo provoked an extra shrinkage of about 27% and 36% versus CGA and Doxo, respectively. Analogously, although CGA was less effective in MG-63 than U2OS cells, all tested combination treatments displayed a greater ability to reduce cell viability even in this other OS model (Figure 2B). In comparison with single agents, co-administration of 200 μM CGA plus 0.1 μM Doxo further affects cell viability in the amounts of 40% and 31% versus CGA and Doxo, individually.

In order to define the nature of drug–drug interaction, CompuSyn analysis was applied to calculate combination indexes (CIs). Based on the median-effect equation, the Chou–Talalay method easily discriminates among additive (CI = 1), synergism (CI < 1), and antagonism (CI > 1) effects when two or more compounds are combined together [30].

Employing the obtained MTT data, CIs were defined for all tested conditions in both U2OS and MG-63 cells. Whilst a moderate synergism came out at very low concentrations in U2OS, a more consistent outcome was revealed when higher doses, affecting 60% and 90% of cells, were employed (Figure 2C). The MG-63 Fa-CI plot suggests a stronger synergism in every experimental condition assessed, even at very low doses instead (Figure 2D).

Subsequently, using 200 μM CGA and 0.1 μM Doxo as effective working dosages, we performed time-course experiments intended to establish the relative impact on cell growth. Although both compounds were already effective in reducing cell number, combination treatment affected tumor doubling to a greater extent (Figure 2E,F). Compared with Doxo alone, co-treatment further decreased cell growth of approximately 15% after 48 h in OS cells, and a similar trend was maintained 24 h later. Regarding CGA instead, a pronounced and different outcome was detected between these two models. Specifically, an additional 39% and 60% of cell number reduction was detected after 48 h of treatment with both CGA and Doxo in U2OS and MG-63, respectively.

Taken together, these findings suggest that combination treatment CGA plus Doxo can synergistically affect cell viability and growth in OS cells.

### 2.3. Co-Administration CGA plus Doxo Further Reduces the Clonogenic Potential in OS Cells

Although quite challenging, targeting cancer stem cells represents an effective therapeutic approach to eradicating cancer [31]. Among the different in vitro tumorigenic assays, the clonogenic assay (as referred to colony-forming) has been recognized as a useful tool to monitor the undifferentiated potential in malignancies [32]. Therefore, to further characterize the CGA plus Doxo anticancer-mediated effects in OS, we investigated the consequences of combination treatment on colony-forming ability.

We previously described that CGA strongly decreases the clonogenic potential of both U2OS and MG-63, whereas several findings report the Doxo ability to restrict colony formation in different cancer models, including OS [23,33].

**Figure 3:**
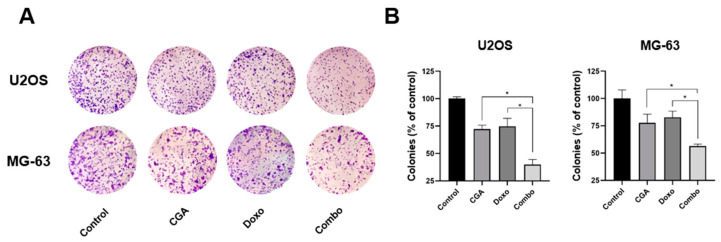
Estimation of CGA, Doxo, and CGA plus Doxo induced colony-forming ability in OS cells. U2OS and MG-63 were treated and not (control) with 20 μM (U2OS) or 40 μM (MG-63) CGA, 0.005 μM Doxo, and CGA plus Doxo (combo); thereafter, clonogenic ability was assessed by crystal violet staining. Representative experimental images are displayed in panel (**A**). Quantification analysis from three different experiments has been carried out and reported in panel (**B**). * *p* < 0.05.

Here, we supplemented the mentioned OS cells with a very low concentration of both CGA and Doxo for 10 days before staining the newly formed colonies with crystal violet. As intentionally designed, the employment of small doses of both CGA and Doxo had a restricted impact on OS colony-forming ability (Figure 3A). Interestingly, the combination of CGA plus Doxo dramatically reduces the OS clonogenic potential, affecting both colony number and size. Quantification analysis further confirmed differences among the experimental groups (Figure 3B).

Altogether, these data indicate that combination CGA plus Doxo provokes a longer-lasting reduction in clonogenic potential compared with single-agent administration.

### 2.4. CGA Enhances Doxo-Mediated Cytotoxic Effects in OS Cells

Since both compounds have been reported to induce cell death in OS, we speculated that combination treatment CGA plus Doxo could enhance single-agent cytotoxicity [23,24,34]. To address this hypothesis, we firstly discriminated living from dead cells using Trypan Blue as the exclusion dye.

**Figure 4:**
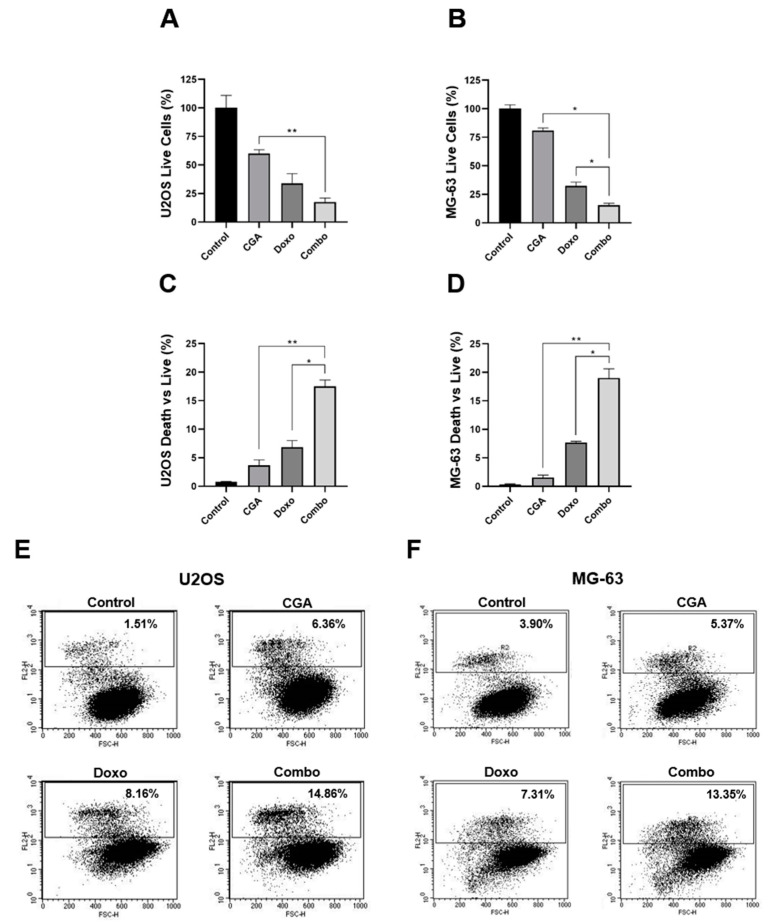
Investigation of CGA, Doxo, and CGA plus Doxo induced cell death in OS cells. U2OS and MG-63 were treated and not (control) with 200 μM CGA, 0.1 μM Doxo, and CGA plus Doxo (combo) for 48 h. Subsequently, living and dead cells were discriminated either by Trypan Blue (**A**–**D**) or by PI assay (**E**,**F**). Representative experimental dot-plots are shown in figure. Living cell number, as well as dead vs. live ratio, is expressed in percentage as mean ± SD. * *p* < 0.05, ** *p* < 0.01 by an unpaired two-tailed *t*-test. Experiments shown in the figure were repeated four times.

A differential cell count highlighted considerable changes between alive and dead cells within the experimental groups, especially when CGA and Doxo were administrated simultaneously. Selective analysis of living cells revealed a 40% and 66% reduction in U2OS when exposed to 200 μM CGA and 0.1 μM Doxo for 48 h, respectively. Considerably, CGA plus Doxo further decreased the alive portion of approximately 16% vs. Doxo and 43% vs. CGA alone (Figure 4A).

Looking at dead cells, we detected a slight increase in response to CGA (+3%) and a more consistent accumulation with Doxo (+7%). Remarkably, the dead vs. live ratio drastically rose by up to 17% in the presence of CGA plus Doxo, doubling the Doxo percentage (Figure 4C). A similar tendency was also achieved in MG-63, where CGA plus Doxo co-treatment reduced living cells and intensified death occurrence compared with a single agent, simultaneously (Figure 4B,D).

To further corroborate this assumption, we also performed FACS-based Propidium Iodide (PI) analysis aimed at defining the cell death contribution to CGA plus Doxo co-administration. Applying an identical experimental setting, PI uptake exhibited a marked increase in positive cells as a consequence of concomitant administration of CGA and Doxo. Precisely, moving the percentage of PI-positive cells from 8.16% to 14.86%, CGA potentiated Doxo-mediated cytotoxic effects in U2OS (Figure 4E). Analogously, compared with anthracycline alone, a six-point increment was detected in MG-63 when CGA and Doxo were administered concurrently (Figure 4F).

Collectively, this evidence strongly supports that CGA reinforces Doxo-mediated anticancer properties, promoting cell death induction in OS cells.

### 2.5. Combination CGA plus Doxo Improves Caspase-3 and PARP Activation in OS Cells

Depending on the dosage applied, Doxo can destroy tumor cells through multiple death mechanisms, including mitotic catastrophe and programmed cell death [35]. Similarly, in most cancer studies in which CGA exhibits cytotoxic properties, apoptosis constitutes one of the main signalings involved in [21,36,37,38].

**Figure 5:**
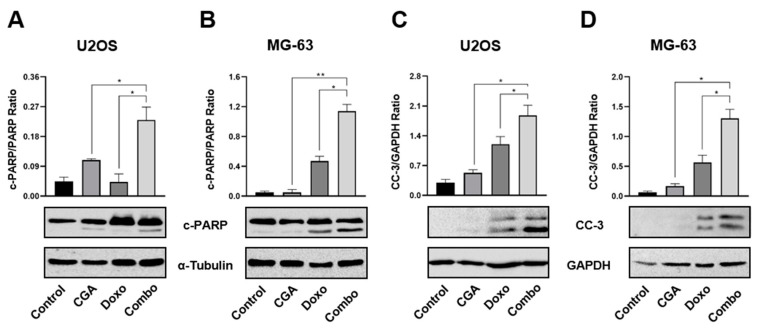
Evaluation of CC-3 and PARP activation in response to CGA, Doxo, and CGA plus Doxo in OS cells. U2OS and MG-63 were exposed and not (control) to 200 μM of CGA, 0.1 μM of Doxo, and CGA plus Doxo (combo) for 48 h. Whole extracts were prepared and then samples were analyzed by Western blotting for PARP (**A**,**B**) or CC-3 (**C**,**D**) protein levels. GAPDH and α-Tubulin were employed as internal loading controls. Representative Western blotting films were shown in the figure together with the relative densitometric analysis obtained from at least three biological replicates. * *p* < 0.05, ** *p* < 0.01 by an unpaired *t*-test.

In light of the emerging results, which propose CGA as a potential cell death enhancer in Doxo-mediated effects, we assessed the expression levels of two distinct biochemical apoptosis hallmarks, namely cleaved caspase-3 (CC-3) and poly (ADP-ribose) polymerase (PARP). Figure 5A,B clearly show a stronger PARP cleavage in reaction to CGA plus Doxo compared with a single agent in both U2OS and MG-63 cells. These results, together with a concomitant intensification in caspase-3 fragmentation (Figure 5C,D), suggest that CGA could improve Doxo-mediated cytotoxicity via apoptosis induction in OS cells.

### 2.6. CGA Ameliorates Doxo-Mediated Cytotoxicity in H9c2 Cardiomyocytes

**Figure 6:**
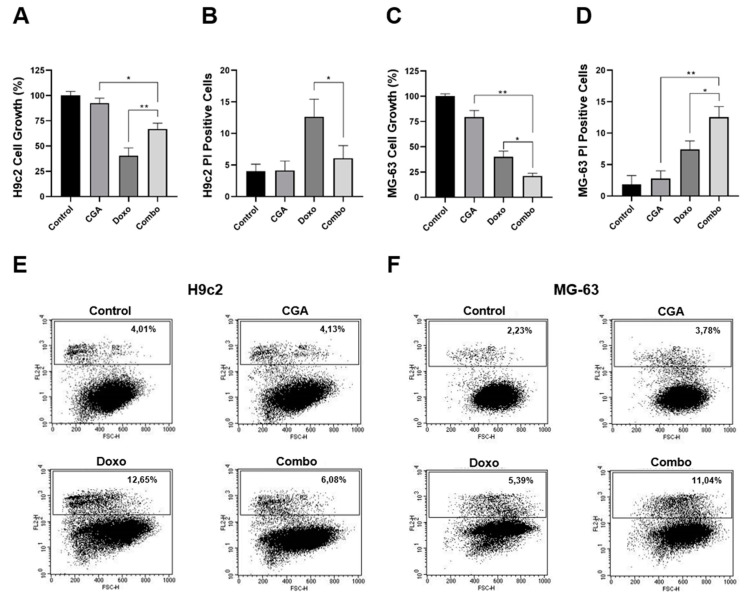
Evaluation of CGA, Doxo, and CGA plus Doxo-mediated effect on cell growth and death in H9c2 and MG-63 cells. H9c2 and MG-63 were treated and not (control) with 200 μM CGA, 0.1 μM Doxo, and CGA plus Doxo (combo) for 48 h. Later, the percentage of cell growth (**A**,**C**) as well as cell death (**B**,**D**) was established. Representative dot-plots are shown in (**E**,**F**). * *p* < 0.05, ** *p* < 0.01 by an unpaired two-tailed *t*-test. Experiments were repeated thrice.

Apart from eliciting resistance mechanisms, Doxo-mediated cardiotoxicity represents the main limiting factor for its prolonged administration in cancer [9,10]. Therefore, increasing Doxo efficacy should go hand-in-hand with reducing side effects, expressly cardiotoxicity. In order to address the combination impact on Doxo-induced toxicity in cardiac cells, we employed embryonic rat cardiac tissue-derived H9c2 cells as a suitable in vitro model to study the anthracycline-mediated effects [39,40,41,42]. More in detail, 200 μM CGA and 0.1 μM Doxo were supplemented alone and in combination to the growing media of H9c2 and MG-63 cells for 48 h. Interestingly, whilst combination treatment further reduced the cell number and increased the percentage of PI-positive cells in MG-63 compared with Doxo alone, this trend was completely reverted in H9c2 cells (Figure 6). Indeed, although Doxo drastically diminished the cell growth rate, combination treatment ameliorated this collateral effect by approximately 27% (Figure 6A). Moreover, CGA extensively counteracted Doxo-induced cell death, considering that the percentage of PI-positive cells moved from around 12.65% to 6.08% when those compounds were administered together (Figure 6B,E). The employed CGA dosage did not affect both cell growth and death in H9c2 cells instead.

Overall, these findings corroborate CGA as a non-toxic agent in H9c2 cardiac cells and further propose this compound as a potential cardioprotective candidate in Doxo-based chemotherapy.

### 2.7. p44/42 MAPK Impairment Promotes CGA-Mediated Doxo Sensitization in OS Cell

Aberrant expression of the mitogen-activated protein kinase (MAPK) pathway has extensively been reported in several neoplastic disorders [43]. Moreover, higher phosphorylation levels of MAPK are generally associated with poorer clinical outcomes, as well as shorter event-free and overall survival in OS [44,45].

Doxo administration usually promotes p44/42 MAPK activation either as a cell resistance mechanism or as cell death signaling [13,46]. Furthermore, different preclinical studies have lately described the CGA ability to modulate p44/42 MAPK in cancer models, selectively [23,25,47,48,49].

**Figure 7:**
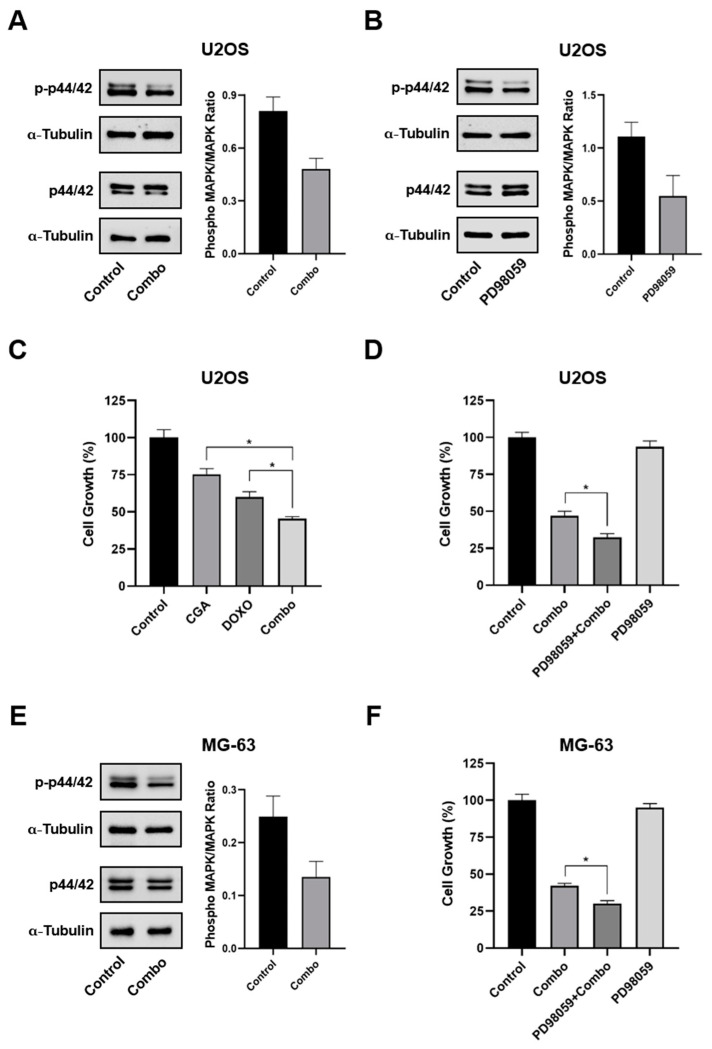
Estimation of MAPK involvement in CGA-mediated Doxo sensitization. U2OS (**A**) and MG-63 (**E**) were treated and not (control) with 300 μM CGA plus 0.2 μM Doxo (combo) for 24 h, and then the relative impact on p44/42 MAPK was assessed by Western blotting. (**B**) Analysis of total and phosphorylated p44/42 MAPK levels following 24 h of exposure to 10 μM PD98059. (**C**) U2OS were treated and not (control) with 300 μM CGA, 0.2 μM Doxo, and CGA plus Doxo (combo) for 24 h, and then the relative cell number in percentage of control was estimated. Combination treatment impact on U2OS (**D**) and MG-63 (**F**) cell growth in an MAPK proficient and hampered background. Three hours pre-treatment with 10 μM of PD98059 preceded and not combo administration. * *p* < 0.05 by an unpaired two-tailed *t*-test. Experiments showed in the figure are the end-result of three distinct biological replicates.

Very interestingly, here we found that CGA plus Doxo downregulated p44/42 MAPK phosphorylation, suggesting a potential involvement in CGA-based combination treatment (Figure 7A,E).

In order to further define the p44/42 MAPK role, we also tested the impact of the selective upstream inhibitor of p44/42 MAPK, namely PD98059, on CGA plus Doxo mediated cell growth effects, firstly in U2OS. As expected, 10 μM of PD98059 was effective in inhibiting p44/42 MAPK phosphorylation (Figure 7B). Nevertheless, although CGA improved Doxo-induced cell growth inhibition, PD98059-mediated p44/42 MAPK impairment enforced this outcome, increasing the inhibition rate by approximately 14% (Figure 7C,D). Comparable PD98059-mediated results were also obtained in MG-63 cells (Figure 7F).

On the whole, these findings propose the p44/42 MAPK pathway as relevantly involved in CGA-mediated Doxo susceptibility.

## 3. Discussion

Increasing Doxo efficacy and mitigating adverse reactions are considered long-standing targets in clinical oncology. To this end, countless Doxo analogs and formulations have been developed and tested over the years [50]. Regrettably, despite the promising results obtained for most of them in preclinical models, Doxil^®^ still remains the only approved Doxo-based therapy by the Food and Drug Administration (FDA) [51].

Among the pharmacological approaches conceived to overcome both Doxo resistance and toxicity, combination therapy surely represents the most-used regimen for treating cancer patients in an advanced state. Nevertheless, whilst there is no doubt about the efficacy of multiple drugs in shrinking the tumor burden and delaying resistance outbreaks, toxicity accumulation from each employed agent still has a deleterious impact on systemic responses [52].

As an attractive source of phytochemical substances, naturally occurring molecules have largely been investigated as chemosensitizers, chemoresistance reducers, or chemotherapeutic protectors in both solid and hematological malignancies [53,54,55].

In line with these fascinating perspectives, here we firstly provide evidence for the employment of CGA as a potential adjuvant agent in Doxo-based chemotherapy, expressively in OS. Being deficient in effective therapy, the combination of neoadjuvant and adjuvant chemotherapy represents the only viable pharmacological approach for OS care [11]. Unfortunately, the mentioned limitations greatly hinder the successful rate of this procedure, calling for more effective strategies.

Interestingly, our results recognize the effectiveness of CGA in enhancing Doxo-mediated outcomes in OS cells. Specifically, although CGA differently affected U2OS and MG-63 behaviors, combination treatment additionally impaired cell viability and growth, as well as colony-forming ability, compared with single agents. As a proof of synergic drug–drug interaction, CompuSyn analysis unveiled CI values less than one in all experimental conditions tested.

Whilst CGA affected Doxo susceptibility in both alive and dead cells, its impact was definitely more pronounced on the non-living portion. This assumption was further supported by the subsequent CC-3 and PARP cleavage analysis, which revealed the highest apoptotic rate when CGA was co-administered with Doxo.

The ability to induce apoptosis has intensely been debated as one of the possible mechanisms by which CGA exerts its antitumor effect in preclinical cancer models, including in OS [21,23,24,36,37]. Specifically, the intrinsic pathway is actively involved in CGA-mediated apoptosis occurrence [21,24,36]. Attenuating mitochondrial membrane potential and reciprocally modulating Bcl-2 and Bax, CGA promotes programmed cell death in A498 human kidney cancer cells, for instance [21]. Moreover, the involvement of the Bax/Bcl-2 pathway has latterly been documented in CGA-mediated apoptosis induction in lung, breast, and hepatoma cancer cells [36,56,57]. Intriguingly, in the only existing study in which CGA potentiates Doxo-mediated cytotoxic effects, Elrazik and colleagues documented a simultaneous engagement of both intrinsic and extrinsic related pathways [26]. Precisely, besides reducing Bcl-2 expression levels, CGA upregulated death receptors such as TRAILR2, TRAIL, Fas, and FasL.

Despite the apoptotic-related mechanisms is currently under investigation in OS cells, initial and preliminary data suggest an implication of the intrinsic pathway in the CGA plus Doxo-mediated apoptosis cell death (data not shown). Nevertheless, the characterization of this aspect, as well as other unrelated cell death mechanisms, will constitute one of the leading aspects for subsequent studies.

Enhancing cancer cytotoxicity is widely considered one of the benchmarks to establish the chances of success for combination therapy. Moreover, combining anticancer with cardioprotective properties could reinforce the significance and translation in clinical.

Importantly, consistent with these perspectives, CGA oppositely affected OS and cardiac cells when co-administrated with Doxo. Whilst CGA enhanced Doxo-induced cytotoxicity in OS cells, amelioration in cell behaviors was observed in H9c2 cardiomyocytes instead. These findings are in agreement with the earliest published study in which CGA is proposed as the best hydroxycinnamic acid for preventing Doxo-induced injuries in Wistar rats’ isolated cardiomyocytes [58]. Comparable results have recently been reported in other cardiomyocyte models by Elrazik and coworkers [26].

Reactive oxygen species (ROS) have been described to play a crucial role in mediating CGA-induced effects on both tumor and cardiac cells [25,26,48]. Depending on cellular models, condition, and doses, CGA and polyphenols can serve either as anti-oxidant or as pro-oxidant agents. In this regard, investigating the CGA consequences in colon cancer, Hou and colleagues recognize the ROS production as a possible explanation for both CGA-induced cell viability inhibition and cell-cycle arrest [48]. This evidence was further supported by the employment of ROS scavenger N-acetylcysteine, which strongly attenuated the CGA-mediated effects. Conversely, in HepG2 human hepatoma cells, CGA promotes ROS elimination, enhancing SOD expression and downregulating NADPH oxidase, concomitantly [28]. Even more intriguing is the outcome in cardiomyocyte models, where, if on one side highest doses seem to have synergic pro-oxidant effects, on the other, reduced concentrations alleviate Doxo-induced mitochondrial oxidative stress [59].

Although the ROS involvement in CGA-induced Doxo sensitivity is currently being studied, here we examined the role of p44/42 MAPK as one ROS-affected cellular pathway [60]. Nevertheless, it is necessary to clarify that as a pleiotropic signaling, p44/42 MAPK is actively involved in cellular homeostasis where it controls nearly every and opposite processes, such as differentiation, survival, proliferation, and death [61].

Given that the ability of modulating p44/42 MAPK has extensively been reported in cancer models for both compounds, we investigated the influence of CGA plus Doxo on this protein [13,25,46,47,48,49]. The CGA plus Doxo combination setting distinctly reduced phosphorylation levels of p44/42 MAPK, suggesting a potential contribution to CGA-mediated Doxo sensitization. Remarkably, in the only existing study in which CGA enhances Doxo-induced toxicity, the inactivation of p44/42 MAPK has also been reported [26].

Despite its controversial role in mediating Doxo effects, different studies recognize MAPK targeting as a potential pharmacological strategy to overcome Doxo resistance in OS. In this respect, Noh and colleagues have shown how, preventing Doxo-induced pro-apoptotic protein expression, p44/42 MAPK blockage facilitates cell death induction in OS cells [62]. More recently, the FDA-approved MEK inhibitor Cobimetinib has been demonstrated to enhance Doxo’s efficacy inhibiting cell growth, survival, and migration in human OS models [63]. Interestingly, the employment of the MEK1/MEK2 inhibitor PD98059 in our experimental conditions promoted combination treatment efficacy.

If on one side our results further support the MAPK role in Doxo-mediated action, at least in OS cells, on the other, they certainly hint at other molecular mechanisms involved in CGA-mediated Doxo susceptibility. That is why additional and more accurate mechanistic experiments must be performed, and we are working on it.

Apart from mechanistic concerns, specific CGA-related issues remain unsolved and should be further addressed before moving to clinical studies. Surely, the restricted solubility and permeability have always hindered the CGA efficacy as a nutraceutical compound. More recently, CGA encapsulation in a nano-sized colloidal delivery vector has been proposed as a possible approach to enhance its antioxidant properties and increase chemopreventive efficacy at an even lower concentration [64]. Moreover, even though an initial CGA Phase I clinical trial has recently been concluded, demonstrating that its injection is safe and well-tolerated in recurrent high grade glioma patients, collecting safety data in a larger statistical sample is absolutely necessary to define the tolerance and pharmacokinetics of this compound (NCT02245204). Limited studies have been conducted even in preclinical models, and thus additional insights must be provided aimed at defining the CGA impact on both cancer and healthy tissues [37,57]. In this respect, we previously addressed the colony-forming ability in both OS and non-tumor mouse embryonic fibroblasts NIH 3T3 [23]. Although the employed cells did not constitute the proper healthy model, no toxicity was observed in response to CGA.

We are conscious that further investigation is absolutely needed to fully recognize the related molecular mechanisms; nevertheless, herein, we recognize the CGA ability to enhance Doxo outcome, proposing this pharmacological approach as an additional chance to treat incurable malignancies such as OS.

## 4. Materials and Methods

### 4.1. Chemical Reagents

Chlorogenic Acid (C3878; Sigma-Aldrich, St. Louis, MO, USA), Doxorubicin (#5927; Cell Signaling Technology, Danvers, MA, USA), PD98059 (#P215; Sigma-Aldrich), 3-(4,5-dimethylthiazol-2-yl)-2,5-diphenyltetrazolium bromide (MTT) (M2128; Sigma-Aldrich), Propidium Iodide (#P4864; Sigma-Aldrich), Trypan Blue solution (T8154; Sigma-Aldrich), and Crystal Violet (C0775; Sigma-Aldrich).

### 4.2. Cell Culture and Experimental Procedures

Human osteosarcoma U2OS and MG-63 cell lines and embryonic rat cardiac tissue-derived H9c2 cardiomyoblasts were purchased by the American Type Culture Collection (Manassas, VA, USA). Cells were routinely maintained in Dulbecco’s Minimum Essential Medium (DMEM) (ECM0728L; Euroclone, Pero, Italy) supplemented with 10% fetal bovine serum (FBS) (ECS0180L; Euroclone) and 100 units/mL penicillin/streptomycin (ECB3001D; Euroclone) at 37 °C in a 5% CO_2_ controlled atmosphere. All the experimental procedures, whose results have already been displayed, consist of three distinct steps, which include seeding, treating, and collecting. Specifically, cells were initially split into an equal number depending on the performed test. After 24 h, the culture medium was replaced with a fresh one containing CGA and Doxo, both alone and in combination. Times and doses are indicated in the “Results” section, as well as in “Figure Legends”. Since CGA and Doxo were reconstituted in ethanol and dimethyl sulfoxide (DMSO), respectively, preliminary tests were carried out with the purpose of addressing the solvent outcome on cell growth and viability. Although no toxic effects were noticed, the same ethanol rate was supplemented in the control group, being that *v/v*% was usually greater or equal to 0.4%. Apart from colony-forming and cell viability assays, trypsin-mediated cell detachment was applied to collect each treatment condition in every procedure.

### 4.3. Cell Viability Assessment

Treated surviving cells were indirectly estimated measuring the relative metabolic activity by non-radioactive and colorimetric MTT assay. In detail, U2OS and MG-63 were seeded in a 96-multiwell plate with a confluence of 1.5 × 10^3^ and 1.3 × 10^3^ cells/well, respectively. Twenty-four hours later, cells were treated with CGA and Doxo, both alone and in combination, as planned by each single experimental procedure. At the designed final point, the growing medium was discarded and wells were washed once in drug-free media before adding 100 μL of detecting solution, in which MTT and culture medium were mixed 1:10. Subsequently, plates were incubated for three hours at 37 °C in a 5% CO_2_ humidified atmosphere. Reduced purple formazan crystals, generated by metabolically active cells, were dissolved, shaking for 30 min at room temperature wells containing 100 μL of Isopropanol-HCl 0.04 N. Finally, quantification analysis was assessed measuring the optical density (OD) at 570 nm (Infinity 200, TECAN, Männedorf, Switzerland).

### 4.4. Cell Growth Evaluation and Colorimetric Exclusion

Using a 6-well plate, U2OS, MG-63, and H9c2 cells were, respectively, seeded at the density of 6 × 10^4^, 4.5 × 10^4^, and 7.0 × 10^4^ per well and then treated in accordance with the experimental design. After reaching the designed end-point, cells were harvested before being manually counted by Bürker’s chamber, employing an inverted phase contrast microscope. By diluting with Trypan Blue (1:1 Ratio), dead cells, formerly stained in blue, were further discriminated from living cells, unstained. Taking an average of no less than two technical replicates, the cell number of each experimental point was defined.

### 4.5. Colony Formation Assay

An identical number of U2OS and MG-63 cells, equal to 1.5 × 10^3^ per well, were plated in a 6-well plate and chronically stimulated for 10 days with the indicated doses of CGA, Doxo, and combination treatment. Once past this period, growing media were thrown away and adherent colonies were fixed and stained for three hours using the all-in-one aqueous solution containing 0.1% crystal violet and 7% ethanol. After incubation, distilled water was used to remove a specific and residual dye. Successively, plates were air-dried and acquired by photographic equipment. Quantitative analysis was performed dissolving colonies-bound crystal violet in 10% acetic acid and establishing the OD at 590 nm.

### 4.6. Propidium Iodide Analysis

As a membrane damage marker, PI was employed to further evaluate drug-induced cell death. Physiologically, an intact phospholipid bilayer completely impedes the PI uptake; nevertheless, massive cell impairment transiently permeabilizes the cell membrane, thus allowing the transport of molecules otherwise not permitted, including PI. Taking advantage of this easy principle, dead cells (PI positive) can be easily discriminated by living ones (PI negative). Briefly, U2OS, MG-63, and H9c2 cells were seeded and treated as previously described in Subparagraph 4.4. After 48 h, cells were collected and stained with 0.4 μg/mL of PI in 1× PBS buffer (ECB4004; Euroclone). The percentage of positive cells was later defined using a BD FACS Calibur analyzer (Franklin Lakes, NJ, USA).

### 4.7. Cell Extracts Preparation

Whole protein extraction was carried out as follows. A number of 3.5 × 10^5^ (U2OS) and 2.6 × 10^5^ (MG-63) cells were allowed to attach to 100 mm plates for 24 h. The following day, drugs were supplemented to the growing media in doses and timings as indicated in the respective experimental procedures (see results for more details). Once harvested, cells were spun-down (5 min at 1500 RPM), rinsed with PBS, and spun-down again. Then, adding 3–5 volume of RIPA buffer (R0278; Sigma-Aldrich), supplemented with both Protease Inhibitor Cocktail (P8340; Sigma-Aldrich) and Phosphatase Inhibitor Cocktail (#5870; Cell Signaling Technology), pellets were lysed on ice for 30 min. Finally, samples were centrifuged (15 min at 14,000 RPM), and the supernatant phase was recovered. The protein content of each sample was quantified by Bradford Assay (39,222; Serva, Heidelberg, Germany).

### 4.8. Western Blotting Analysis

Detection of specific proteins among the experimental series required a proper sample preparation, in which, for each specimen, the Laemmli buffer 2X (S3401; Sigma-Aldrich) was mixed with an equal protein amount before heat denaturation (5 min at 95 °C). Subsequently, loading a protein range from 15 to 30 µg, electrophoresis was performed in sodium dodecyl sulfate polyacrylamide gel (SDS-PAGE). After moving proteins to the nitrocellulose membrane (Amersham Protran Premium; Cytiva, Marlborough, MA, United Stetes), foils were firstly blocked for one hour in no-fat milk (5% *w*/*v*) (A0830; PanReac Applichem, Chicago, IL, USA) and then incubated overnight at 4 °C with specific primary antibodies. The next morning, HRP secondary antibodies, recognizing the related primary species, were applied to the membrane for 1 h at room temperature. Finally, chemiluminescence was detected with the Chemi Doc XRS (Bio-Rad, Hercules, CA, USA) instrument using liteablot chemiluminescent as a substrate (EMP011005; EuroClone). Each incubation step was preceded and followed by three washes in TBS plus 0.05% Tween-20 (TC287; HIMEDIA, Mumbai, India) (T-TBS).

### 4.9. Antibodies

p44/42 MAPK (ERK1/2) (#9102) (1:1000, 5% *w*/*v* BSA in T-TBS), phospho-p44/42 MAPK (ERK1/2) (Thr202/Tyr204) (#9101) (1:1000, 5% *w*/*v* BSA in T-TBS), PARP (#9542) (1:1000, 5% *w*/*v* no-fat dry milk in T-TBS), cleaved caspase-3 (Asp175) (5A1E) (#9664) (1:500, 5% *w*/*v* BSA in T-TBS), α-Tubulin (DM1A) (#3873) (1:500, 5% *w*/*v* milk in T-TBS), GAPDH (D16H11) (#5174) (1:1000, 5% *w*/*v* milk in T-TBS), and goat anti-rabbit IgG HRP-linked (#7074) (1:2500, 5% *w*/*v* milk in T-TBS) were purchased from Cell Signaling Technology. Goat anti-mouse IgG HRP-linked (GtxMu-003-EHRPX.0.05; Immunoreagents Inc., Raleigh, NC, USA) (1:2500, 5% *w*/*v* milk in T-TBS) and goat anti-rabbit IgG HRP-linked (1:2500, 5% *w*/*v* milk in T-TBS) were employed as secondary antibodies for immunoblotting.

### 4.10. Statistical Analysis

Mean ± SD of biological replicates was reported. Analysis of variance (ANOVA) and t-Student’ tests were applied with the purpose of discriminating significant differences among the experimental groups. A value of less than 0.05 was recognized as significant. Image J (NIH, Bethesda, MD, USA) was employed to carry out densitometric analyses.

## Data Availability

The data presented in this study are available on request from the corresponding author.
